# *Kingella kingae* septic arthritis in children: recognising an elusive pathogen

**DOI:** 10.1007/s11832-014-0549-4

**Published:** 2014-01-23

**Authors:** Nicole Williams, Celia Cooper, Peter Cundy

**Affiliations:** 1 Department of Orthopaedics, Women’s and Children’s Hospital, 72 King William Rd, North Adelaide, SA 5006 Australia; 2 Centre for Orthopaedic and Trauma Research, University of Adelaide, Adelaide, SA Australia; 3 Department of Microbiology and Infectious Diseases, Women’s and Children’s Hospital, 72 King William Rd, North Adelaide, SA 5006 Australia

**Keywords:** Infection, Septic arthritis, Polymerase chain reaction, Synovial fluid

## Abstract

**Purpose:**

*Kingella kingae* is an increasingly identified cause of musculoskeletal infections in young children. We report our experience with a recently developed polymerase chain reaction (PCR) method and review the clinical course of children diagnosed with *K. kingae* septic arthritis in a tertiary referral paediatric hospital.

**Methods:**

All positive cases of *K. kingae* identified by PCR analysis of synovial fluid from August 2010 until July 2013 were included. A chart review was undertaken to determine history, presentation and management.

**Results:**

27 Children (14 male, 13 female) had PCR positive synovial fluid samples for *K. kingae* with median age of 19 months (range 4 months to 5 years 3 months). The sites of infection were knee (17 cases), hip (2 cases), ankle (5 cases), shoulder (2 cases) and elbow. The median temperature on presentation was 37.1 °C, median peripheral white blood cell count 12.4 (9.9–13.8) × 10^9^/L, erythrocyte sedimentation rate 55 (48–60) mm/h and C-reactive protein 24 (8–47) mg/L. The median synovial fluid white cell count was 21.8 (16.7–45.0) × 10^9^/L. Routine cultures identified *K. kingae* in only two synovial fluid samples. Two samples were additionally positive for *Staphylococcus aureus*.

**Conclusions:**

*Kingella kingae* is a significant cause of septic arthritis in young children. The authors recommend maintaining a high index of suspicion in young children presenting with joint inflammation, especially if indices of infection are mild. It appears likely that children historically treated with antibiotics for “culture negative” septic arthritis were infected with *K. kingae*. PCR techniques for detection of *K. kingae* should be encouraged.

## Introduction

*Kingella kingae* is being increasingly recognised as a pathogen responsible for musculoskeletal infections in the paediatric population [1]. It has been proposed that it is the major bacterial cause of osteoarticular infections in children less than 4 years of age [2], and was responsible for a reported outbreak of musculoskeletal infections in toddlers attending a single child care centre [3, 4]. Despite this, there is not yet widespread knowledge among the medical community of the existence of this organism.

*Kingella kingae* is a ß-haemolytic aerobic and facultative anaerobic Gram-negative bacillus that is part of the normal commensal flora of the pharynx in young children [1]. It is rarely identified in children less than 6 months, but has been shown to colonise 9–12 % of children aged 12–24 months, with carriage rates decreasing thereafter [5]. Musculoskeletal infections may follow a period of bacteraemia associated with a breach of the respiratory epithelium. In support of this theory, a history of preceding upper respiratory illness or pharyngeal ulceration is often reported [6, 7].

The classic signs and symptoms of septic arthritis (fever, anorexia, general irritability, a painful, swollen joint with decreased range of motion and inability to bear weight if the lower limb is involved) [8] may be less pronounced or absent with *K. kingae* infections [2, 9] than with the more commonly recognised *Staphylococcus aureus*.

The true incidence of paediatric *K. kingae* septic arthritis is unknown, as the clinical presentation may mimic transient synovitis or rheumatologic conditions and the organism is often not detected by routine culture techniques [10].

Standard laboratory culture techniques with specimens inoculated onto solid media rarely yield a growth of *K. kingae* [1]. Improved rates of detection from 63 to 100 % may be seen when synovial fluid specimens are collected in aerobic blood-culture vials, but the sensitivity of this method is highly dependent on the system used [11, 12]. Detection of the organism approaches 100 % when polymerase-chain reaction (PCR) techniques are used [1, 13].

This study aims to report the presentation, clinical course and management of children diagnosed in a paediatric tertiary referral centre with *K. kingae* septic arthritis, and to present our experience with the use of a newly developed PCR method for identification of *K. kingae* infection.

## Materials and methods

Approval for the study protocol was obtained from the hospital Human Research Ethics Committee (HREC/12/WCHN/533A).

The setting is a paediatric tertiary referral hospital treating children 18 years of age and younger. Since August 2010, an in-house laboratory at our institution has performed PCR detection for *K. kingae.* The assay technique described by Lehours et al. [13] uses real-time PCR platform targeting the toxin-encoding gene *rtxA* of *K. kingae*. For the majority of this study period, the assay was not considered routine, and was performed for diagnostic and validation purposes on synovial fluid samples that arrived at the specific laboratory during standard working hours only. Synovial fluid samples collected out of hours were sent to an off-site facility where *K. kingae* PCR is not performed.

There is no clinical pathway for children with signs and symptoms of joint inflammation at our institution, with cases referred to the rheumatology or orthopaedic team at the discretion of the assessing emergency room doctor, and with subsequent management determined by the admitting specialist.

A chart review of all synovial fluid PCR positive cases of *K. kingae* identified between August 2010 and July 2013 was undertaken to record demographics (age, gender), history of upper respiratory or other infective symptoms, clinical signs and blood parameters on presentation and synovial fluid and blood culture results, surgical intervention and antibiotic treatment. The demographics and laboratory results for PCR negative cases are presented for comparison. Unless otherwise stated, data are presented as median and interquartile range (IQR). Logistic regression was used to determine predictors of a PCR result positive for *K. kingae*.

## Results

In the 3-year study period, a total of 68 synovial fluid samples underwent PCR testing for *K. kingae*, of which 27 children (14 male and 13 female) had samples that were *K. kingae* positive. The demographics and laboratory results of these cases and the 41 who returned negative *K. kingae* PCR results are summarised in Table 1. The total number of synovial fluid samples collected at our institution over the study period, including those sent off-site that did not undergo PCR detection for *K. kingae*, was not able to be determined. The median age of the children with positive results was 18.6 months, with the youngest child 4 months of age and the oldest 5 years and 3 months. This differed significantly from the median age of 4.8 years (range 7 months–16.2 years) for *K. kingae* PCR negative cases. The sites of *K. kingae* infection were knee (17 cases), hip (2 cases), ankle (5 cases), shoulder (2 cases) and elbow.

**Table 1:** Summary of *K. kingae* PCR positive and negative cases

	*K. kingae* PCR positive (*N* = 27)	*K. kingae* PCR negative (*N* = 41)	*p* value*
Gender			0.075
Male	14 (51.8 %)	30 (73.2 %)	
Female	13 (48.2 %)	11 (26.8 %)	
Age (years)	1.6 (1.2–1.9)	4.8 (2.9–11.4)	0.001
Joint involved			
Ankle	5 (18.5 %)	3 (7.3 %)	0.08^a^
Elbow	1 (3.7 %)	4 (9.8 %)	
Hip	2 (7.4 %)	10 (24.4 %)	
Knee	17 (63.0 %)	24 (58.5 %)	
Shoulder	2 (7.4 %)	0 (0 %)	
White cell count (×10^9^/L)	12.4 (9.9–13.8)	11.2 (8.2–15.1) (*N* = 40)	0.2
CRP (mg/L)	24.0 (8.0–47)	46.5 (10.5-88.5) (*N* = 40)	0.06
ESR (mm/h)	55 (48–60) (*N* = 13)	42 (25–74) (*N* = 27)	0.86
Fluid WCC (×10^9^/L)	21.8 (16.7–45.0) (*N* = 11)	11.7 (3.2–18.0) (*N* = 19)	0.45
Synovial fluid culture			
Negative	22 (81.5 %)	29 (70.7 %)	
*S. aureus*	2 (7.4 %)	8 (19.5 %)	
Gram negative bacillus	1 (3.7 %)		
*K. kingae*	2 (7.4 %)		
*MRSA*		2 (4.9 %)	
*Haemophilus infuenzae*		1 (2.4 %)	
*Strep.*species		1 (2.4 %)	

Data presented as median (IQR)/count (percent)

CRP C-reactive protein, ESR erythrocyte sedimentation rate, WCC white cell count, MRSA methicillin-resistant *Staphylococcus aureus*

*Logistic regression

^a^Fisher’s exact test

The median duration of limb symptoms prior to initial presentation to the Emergency Department was 3 days (range 12 h to 5 days). Five of the children were initially sent home from the Emergency Department with a diagnosis of suspected transient synovitis. Three of the children re-presented to the Emergency Department on more than one occasion. Orthopaedic opinion was ultimately sought following worsening of objective clinical signs on subsequent visits.

Ten of 16 walking-age children with septic arthritis of the knee and three of five walking-age children with ankle involvement were able to walk with a limp. The one walking-age child with hip infection was unable to bear weight. Twelve children had preceding upper respiratory symptoms, with one undergoing a course of amoxycillin for tonsillitis and otitis media. Three children had preceding diarrhoeal illness. One child was receiving prednisolone for hand, foot and mouth disease; there was one case of concomitant herpes simplex stomatitis and one case of oral candidiasis. Nine children had no preceding upper respiratory or gastrointestinal symptoms.

The median temperature on initial presentation was 37.1 °C, peripheral blood white cell count 12.4 (9.9–13.8) × 10^9^/L, erythrocyte sedimentation rate 55 (48–60) mm/h and C-reactive protein 24 (8–47) mg/L. Blood cultures were performed in 22 of the cases, with 21 failing to culture any organisms. One sample was positive for *Staphylococcus epidermidis*, its presence interpreted as a contaminant.

The provisional diagnosis on admission was recorded as presumptive septic arthritis in nine cases, reactive arthritis in two cases and osteomyelitis in three cases. The remaining children with joint pain or joint swelling were admitted for investigation. Plain radiography of the affected joint was performed on all 27 children, 23 children also underwent ultrasound, and a bone scan was performed in one child with a negative joint ultrasound for knee effusion. Four children underwent magnetic resonance imaging (MRI), which required a general anaesthetic.

All synovial fluid samples were collected under sterile conditions in the Operating Room under general anaesthetic, or the Radiology Department or Emergency Department under local anaesthetic or conscious sedation. Eleven cases of septic arthritis of the knee underwent arthroscopic irrigation. Open arthrotomy was performed on three knees, one ankle, one elbow and two hip joints. Four ankles, two shoulders and three knees underwent needle arthrotomy and irrigation with normal saline. One girl aged 5 years and 3 months with knee involvement underwent repeat arthroscopic washout when clinical and laboratory parameters failed to improve after initial surgery and 4 days of intravenous ceftriaxone.

Synovial fluid samples collected from eight knees, one shoulder and two ankles were suitable for cell count, and these returned a median synovial fluid white cell count of 21.8 (16.7–45.0) × 10^9^ per litre. All cases were PCR positive for *K. kingae*. 22 cases had no bacteria seen on Gram stain, and had no growth on culture. The decision whether to inoculate blood culture bottles with synovial fluid rested with the doctor collecting the sample, and the BacT/Alert system was used. We do not have information on the number of samples that were treated in this manner. *Kingella kingae* was cultured and identified from one sample sent in a Paediatric BacT/Alert vial and from one synovial fluid swab. These culture results were only available following the patients’ discharges, with the organism already identified on PCR. A Gram-negative bacillus was cultured from a synovial fluid sample sent in a specimen pot, only with the organism unable to be identified by the automated biochemical identification system. One shoulder and one hip demonstrated Gram-positive cocci and were culture positive for *S. aureus*.

The Infectious Diseases Department was consulted for all cases and determined the choice of antibiotic. Intravenous therapy with flucloxacillin (50 mg/kg 6 hourly), cefazolin, ceftriaxone, cefotaxime, benzyl penicillin, or a combination was used. Intravenous antibiotics were changed to oral antibiotics following improvement in clinical and laboratory parameters. Oral courses of flucloxacillin, cephalexin or amoxicillin ± clavulanic acid were prescribed and patients were discharged home when tolerating oral therapy. The median length of stay was 6 days (range 2–28).

## Discussion

Since evaluating a specific PCR assay, we have treated 27 young children with a diagnosis of *K. kingae* septic arthritis. It is being increasingly acknowledged that the prevalence of paediatric musculoskeletal infection caused by this organism is likely to have been grossly underestimated, and *K. kingae* has now been identified as a significant pathogen in cases of spondylodiscitis [14], septic arthritis and osteomyelitis [4, 15, 16]. This paper provides evidence of the significance of this organism in a paediatric population, as well as the continuing difficulties identifying cases due to the subtle clinical presentation, equivocal blood parameters and poor yield on routine culture techniques.

Clinical prediction algorithms developed using retrospective cases series and validated prospectively have been used to distinguish between septic arthritis and transient synovitis of the hip (the Kocher criteria) [17, 18]. Demographics and laboratory results for the cases in our institution with negative PCR for *K. kingae* are presented for comparison, but as negative cases will likely represent a heterogenous group of culture negative and culture positive septic arthritis, transient synovitis and rheumatological conditions, we are unable to draw firm conclusions. *K. kingae* cases were overall younger, more likely to be female, less likely to involve the hip and with lower C-reactive protein (CRP) than other pathologies that led to synovial fluid analysis in our series.

Symptoms and blood results were less florid than might be expected with septic arthritis caused by *S. aureus*, contributing to a delay in diagnosis in five patients initially discharged home from the emergency department. Septic arthritis was the admitting provisional diagnosis in only nine of the 27 cases, and it is possible that with increased awareness of the existence and presentation of *K. kingae* septic arthritis, investigations such as bone scans and MRI under general anaesthetic could be avoided.

Eighteen of the 27 children in the current series had a preceding illness, with the oro-pharyngeal tract involved in 15 cases. It is important to recognise that arthralgia in this scenario may support a diagnosis of *K. kingae* [6, 7], rather than transient synovitis.

Synovial fluid samples from two patients cultured *S. aureus* following identification of Gram positive cocci on microscopy. It is possible that this represents super-infection of a joint already infected with *K. kingae* in a similar way that bacterial super-infection can follow viral upper respiratory infections. To our knowledge, this has not been previously reported with *K. kingae*.

In addition to the standard limitations of a retrospective audit of this nature, a further limitation of this study is that for the PCR to be performed in our institution, samples were required to be referred to a specific laboratory that only operates during standard working hours. We are therefore unable to comment on the incidence of *K. kingae* in our population, as the laboratory was not routinely referred samples out of hours and may not have been referred all samples in hours, with potential for cases of *K. kingae* to remain undiagnosed. The increasing recognition of *K. kingae* as a pathogen has now led to a change in practice, with retention of a separate synovial fluid sample for PCR in addition to samples sent for regular analysis out of hours. Previously undiagnosed cases may have undergone an abortive course [1], or may have undergone treatment as presumed septic arthritis. This is highly likely, as synovial fluid samples in all cases of septic arthritis are Gram stain positive in only 30–50 % of cases and culture positive in 50–80 % [8], and *K. kingae* is effectively treated by a wide range of antibiotics given empirically to young children [12, 19]. The fact that five children in our series had more than one presentation to the Emergency Department and one child required two arthroscopic knee washouts confirms that infection with *K. kingae* should not be considered a benign, self-limiting condition [1].

The detection and identification of *K. kingae* in synovial fluid has improved significantly since the development of PCR techniques [13, 14, 20]. The specificity of these assays relies on the ability of the PCR to generate only the expected amplification product [21], and a number of gene targets have been tested [13]. The PCR technique used in this study targets the *rtxA* toxin gene and has been demonstrated to be 100 % specific for *K. kingae* [13], with a sensitivity ten times higher than a previously reported technique [22]. Additional benefits of PCR are that bacterial genetic material is still detected in synovial fluid up to six days after initiation of antibiotic therapy [20] and identification is often achieved within 24 h [1, 13]. It has been suggested that PCR analysis of blood for *K. kingae* may obviate the need for more invasive sample collection such as in cases of spondylodiscitis [14]. This approach has not been adopted in our institution due to concerns regarding the specificity of this method to confirm an osteoarticular cause for the bacteraemia.

It appears likely that many children treated in the past with antibiotics for culture negative (by standard laboratory techniques) septic arthritis were, in fact, suffering from *K. kingae* bacterial sepsis. With increasing awareness and improved laboratory techniques including the use of PCR, *K. kingae* will continue to declare itself as a significant cause of septic arthritis in young children. Prospective studies will shed further light on the condition. The authors recommend that a high index of suspicion of *K. kingae* infection be maintained in children 5 years of age and younger presenting with joint pain, a limp or other suggestive clinical features, especially if these indices are mild.
